# Program Staff Perspectives on Virtual and Hybrid Volunteering in Senior Companion and Foster Grandparents Programs

**DOI:** 10.1093/geroni/igaf122.3831

**Published:** 2025-12-31

**Authors:** Cynthia Cushing, Jennifer Crittenden, Rachel Coleman, Nathan Tarbox

**Affiliations:** University of Maine, Orono, Maine, United States; University of Maine Center on Aging, Orono, Maine, United States; University of Maine Center on Aging, Orono, Maine, United States; University of Maine, Orono, Maine, United States

## Abstract

Volunteering has been linked to improved health and well-being, increased socialization, and reduction of social isolation among older adults. During the COVID-19 pandemic, virtual volunteering was introduced to existing volunteer programs as a strategy to retain volunteers while staying physically distanced. Semi-structured Zoom-based interviews were conducted with 18 program directors across the U.S. within two predominantly in-person volunteer programs, the Senior Companion Program (SCP) and Foster Grandparent Program (FGP). Interviewees (N = 18) included eight SCP, eight FGP, and two representing both programs. Qualitative analysis yielded 16 parent codes within the data. Findings suggest COVID-19 was an impetus for increased VV use in volunteer programs. Post-pandemic, the study found the majority of volunteering has returned to in-person activities, with increased technology adoption for administrative functions with volunteers. Interviewees reported that VV has predominantly occurred on a case-by-case basis for individual volunteers that prefer this modality. Program directors expressed mixed views on VV and technology use with older adult volunteers, with some optimistic and open to future opportunities. Technology access, digital literacy, negative attitudes towards technology, lack of funding, staffing, training capacity, and administrative barriers were found to be challenges to implementing and maintaining VV over the long-term.

